# Optimization of the Method for Isolating Bacterial DNA from the Aboveground Part of Lettuce

**DOI:** 10.3390/ijms25158513

**Published:** 2024-08-04

**Authors:** Magdalena Krupka, Agnieszka I. Piotrowicz-Cieślak

**Affiliations:** Department of Plant Physiology, Genetics and Biotechnology, University of Warmia and Mazury, Oczapowskiego Str. 1A, 10-719 Olsztyn, Poland; magdalena.krupka@uwm.edu.pl

**Keywords:** phyllosphere, plant, mechanical–chemical lysis, bacteria

## Abstract

Developing an effective method for isolating bacterial genetic material from plants is a relatively challenging task and often does not yield adequately prepared material for further analyses. Previous studies often overlook connections, primarily focusing on laboratory investigations. With advancements in high-throughput sequencing techniques, we can now revisit and delve deeper into these interactions. Our study focuses on the initial phase of these investigations: genetic material isolation. Extracting bacterial DNA from aboveground plant parts, known as the phyllosphere, poses a significant challenge due to plant-derived contaminants. Existing isolation protocols frequently yield inconsistent results, necessitating continuous refinement and optimization. In our study, we developed an effective isolation protocol employing mechanical–chemical lysis, sonication, and membrane filtration. This approach yielded high-quality DNA at a concentration of 38.08 ng/µL, suitable for advanced sequencing applications. Our results underscore the effectiveness and necessity of these methods for conducting comprehensive microbiological analyses. Furthermore, our research not only lays the groundwork for further studies on lettuce microbiota, but also highlights the potential for utilizing our developed protocol in investigating other plants and their microbiomes.

## 1. Introduction

The “One Health” concept describes the interactions between humans, animals, plants, and their environment, emphasizing the interdependence of these components for public health maintenance [1]. Microorganisms are a key element of this concept, and act as a bridge between these components, impacting the health of all three domains of life [2]. Soil serves as the primary environmental habitat for microorganisms, forming the basis of the microbiological ecosystem [3]. However, the greatest threat to soil bacteria comes from antibiotics and their metabolites, which are routinely detected in agricultural soils. Even low concentrations of antibiotics in soils can lead to declines in certain bacterial groups and induce antibiotic resistance, posing a serious public health threat. Projections indicate that, by 2050, antibiotic resistance will become the leading cause of death in the human population [4].

To date, most research has focused on the spread of antibiotic resistance in hospital environments [5]. However, the “One Health” concept has highlighted the presence of antibiotic-resistant strains in other environmental components, including food products. Most studies on microorganisms and antibiotic resistance genes (ARGs) transmitted through the food chain pertain to animal-derived products [6,7,8,9,10,11]. Changes in dietary habits and the popularity of vegan and vegetarian diets have made raw plant products an important element of the human diet [12]. Unfortunately, the role of fresh vegetables and fruits in the spread of ARGs is still marginalized [5].

The concept of edible plant microbiota is relatively new and assumes that fresh vegetables and fruits can be sources of microorganisms, both pathogenic and beneficial, that colonize the human body [13]. The Centers for Disease Control and Prevention (CDC) in the USA indicates that 46% of foodborne infections are associated with the consumption of fresh vegetables and fruits [14]. On the other hand, some beneficial bacteria have representatives in both the gut and plant microbiomes, suggesting that bacteria colonizing plants may perform beneficial functions in the human body [15].

Understanding the composition and function of the microbiota of fresh vegetables and fruits is therefore important from both consumer and public health perspectives. The aboveground parts (phyllosphere) of lettuce, one of the most commonly consumed raw vegetables, are colonized by numerous microorganisms [16]. Studies on the phyllosphere of plants, particularly those growing in polluted environments, are still rare in the literature.

Only a small fraction of the microorganisms colonizing plants can be cultured on traditional media [17]. The development of high-throughput sequencing methods allows for both the identification and study of complex microbial populations [18]. Although sequencing methods are highly specific, the material being studied must not be contaminated or degraded. Eliminating contaminants is especially important for samples with low microbial content, such as those from the phyllosphere [19]. Therefore, developing a repeatable and optimal method for isolating bacterial DNA from leaves is crucial.

Although many protocols for the separation and isolation of DNA from the phyllosphere are available, they are not consistent [20]. A typical isolation protocol consists of several stages: the preparation of plant material, the separation of bacterial cells, the lysis of bacterial cells, and DNA purification [21]. Sample preparation involves shaking plant fragments with a PBS solution [22] or NaCl [23]. Bacterial cells are then collected by centrifugation [24] or membrane filtration [22,25]. Some protocols recommend cutting the membrane into small fragments, while others use the entire membrane for isolation. Most available protocols for isolating DNA from phyllosphere bacteria recommend using commercial kits for isolating genomic DNA from soil. These kits mainly use enzymatic or mechanical lysis [21] and differ in their effectiveness in lysing specific microorganisms [19].

Differences between sample preparation and isolation protocols can introduce errors in the taxonomic analysis of the phyllosphere microbiome, and even cause the loss of certain bacterial groups in a sample. Moreover, a large portion of the available protocols pertain to ready-to-eat plant products, which undergo preparation processes before sale that may lead to the transfer of ARGs to the final product [5], and thus do not reflect real environmental conditions.

The aim of this study was to compare protocols for the separation of bacteria and the isolation of bacterial DNA from the phyllosphere of lettuce grown in soil collected from a field. Developing a repeatable and effective method for isolating DNA from plant samples is the first and most important step in molecular studies of microorganisms colonizing lettuce. Understanding the lettuce microbiota can provide significant insights into plant–microbe interactions and potential human health impacts, in line with the principles of the “One Health” concept.

## 2. Results and Discussion

Sequencing methods require genetic material of appropriate quantity and quality [26]. Although advancements in next-generation sequencing (NGS) methods allow for accurate results from small amounts of genetic material, a DNA concentration above 10 ng/µL is recommended to ensure optimal performance [27]. One of the most influential factors affecting the quantity and quality of the obtained material is the isolation method [28]. Isolating bacterial DNA from environmental samples, including the phyllosphere, presents particular challenges due to the presence of contaminants that can interfere with PCR methods [21,29].

The choice of an appropriate protocol is crucial for isolating genetic material with the correct quantitative and qualitative parameters, enabling further analysis. Most available protocols for isolating DNA from phyllosphere bacteria utilize mechanical lysis. However, Yang et al. [21] indicate that mechanical lysis can cause fragmentation and a subsequent loss of genetic material. On the other hand, using milder enzymatic lysis carries the risk of introducing contaminants from plant material [21], significantly affecting the quality parameters of the isolated DNA.

Our results indicate that enzymatic lysis does not yield DNA of adequate quality, as demonstrated in experiments 1–12, where the OD 260/280 ratios ranged from 1.2 to 1.54 (Table 1). Similar results were obtained by Yang et al. [21] when isolating DNA from the phyllosphere of alfalfa and by Baturo-Cieśniewska et al. [30] when isolating genomic DNA from soybean rhizosphere. These findings underscore the limitations of enzymatic lysis in producing high-quality DNA from phyllosphere samples.

Another parameter for assessing the quality of isolated genetic material is the A260/230 ratio, which should range between 1.8 and 2.2 [31]. In our study, this parameter was very low (0.57–1.11 in experiments 1–12 with enzymatic lysis), suggesting contamination of the isolated genetic material. The highest DNA concentrations (10.3 and 11.5 ng/µL) were obtained in experiments 8 and 9, where samples were prepared through sonication and membrane filtration with an initial material amount of 50 g. However, these samples did not meet quality standards (OD 260/280 below 1.8) (Table 1). Thus, despite multiple sample-preparation steps, enzymatic lysis methods are not suitable for isolating DNA from the phyllosphere microbiome.

Yang et al. [21] demonstrated that mechanical lysis allows the obtainment of genetic material with good quality parameters, although the DNA yield is lower compared to enzymatic lysis. Our results show that using mechanical–chemical lysis produced genetic material with better quality parameters compared to enzymatic lysis (Table 1). The purity parameters ranged from 1.6 to 1.9 (A260/280) and 0.91 to 1.87 (A260/230). DNA was obtained in a range of concentrations from 5.4 ng/ µL to 38.08 ng/µL (Table 1). These findings indicate that the cell lysis method is not the only factor determining the amount of genetic material obtained. The phyllosphere, compared to the rhizosphere, is an environment with a lower abundance of microorganisms [27]. Therefore, obtaining large amounts of DNA requires additional sample-preparation methods.

One approach that significantly increases the concentration of isolated DNA is sonication. Sonication is widely used for separating bacterial cells from soil [32]. Most phyllosphere microbiome isolation protocols also recommend sonication for separating bacterial cells from leaves [27,33]. Our results indicate that sonication yields higher DNA concentrations, although it is not the sole factor for obtaining the appropriate amount of genetic material. The next step involves collecting and concentrating bacterial cells. Our results show that membrane filtration yields more DNA than cell collection by centrifugation (Table 1).

The preparation method of the membrane for isolation is also crucial. Both cut [34] and whole membranes [22] are used in phyllosphere DNA isolation protocols. Using membranes cut into small fragments results in significantly higher DNA yields (38.08 ng/µL) compared to whole membranes (24.29 ng/µL) (Table 1). This suggests that the increased surface area of cut membranes enhances the efficiency of DNA recovery. The best quantitative and qualitative results were obtained using mechanical–chemical lysis, sonication, and membrane filtration (experiments 20 and 21), along with increasing the amount of initial material to 50 g. This combination allowed for the isolation of DNA with the desired quality and concentration, demonstrating that integrating multiple preparation steps is essential for optimizing DNA extraction from phyllosphere samples.

**Figure 1:**
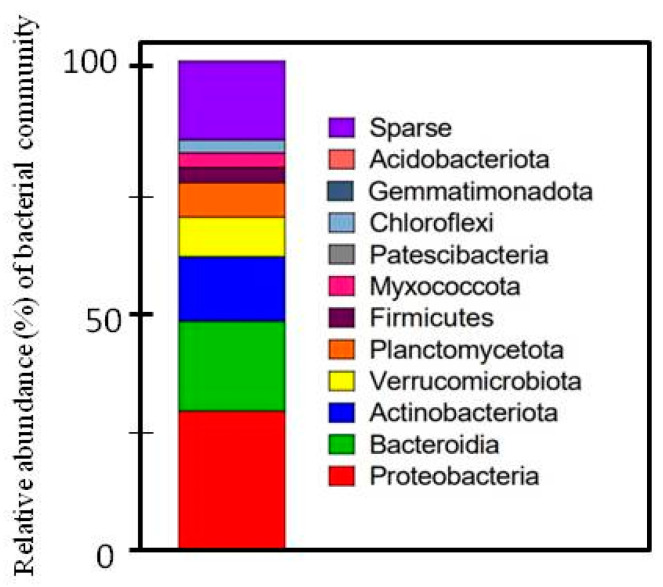
Relative abundance (%) of bacterial community. Taxons that made up less than 5% of total classified reads were grouped and labeled as “Sparse”.

To further confirm the quality of the isolated DNA and its utility in molecular studies, 16S rRNA gene sequencing was performed. This procedure involved the PCR amplification of a selected region of the 16S rRNA gene, followed by sequencing of the resulting products. The DNA with the best quantitative and qualitative parameters (experiment 21, Table 1) was used for 16sRNA sequencing. The sample was prepared using sonication and membrane filtration (cut membrane), and the amount of tissue was 50 g (Table 2). In total, we obtained 84,068 raw-read pairs from the sequencing run. Of these sequences, 99.85% were successfully assigned to the bacterial kingdom, demonstrating the high specificity and effectiveness of our DNA extraction and sequencing protocols in targeting bacterial DNA. This high assignment rate underscores the robustness of our method in isolating bacterial genetic material while minimizing contamination from non-bacterial sources. The relative abundance of bacterial communities is presented in Figure 1.

**Figure 2:**
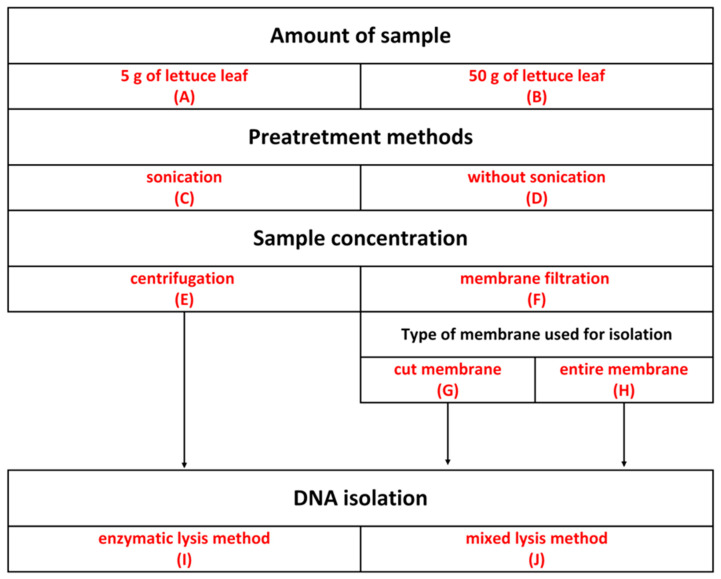
Overview of experimental steps of bacterial DNA isolation from lettuce phyllosphere.

In our study, the most abundant groups were Proteobacteria, Bacteroidetes, Actinobacteria, and Verrucomicrobiota (Figure 2). Meanwhile, Trivedi et al. [35] indicated that plants are most commonly colonized by bacteria belonging to Proteobacteria, Bacteroidetes, Firmicutes, and Actinobacteria. Moreover, we identified 84 reads (0.2006%) attributed to *Lactuca sativa* L. chloroplast DNA. This low percentage indicates that our method effectively limited the co-extraction of chloroplast DNA. Chloroplast contamination is a common issue in plant-associated microbiome studies, as plant tissues often contain high amounts of chloroplast DNA, which can interfere with the accurate profiling of bacterial communities [36]. The minimal presence of chloroplast DNA in our results highlights the precision of our extraction process, confirming that the majority of the isolated DNA was of bacterial origin. The reduction in chloroplast DNA contamination is particularly significant because it allows for a more accurate representation of the bacterial community within the phyllosphere. Chloroplast DNA can overshadow bacterial sequences, leading to skewed or incomplete data. By minimizing chloroplast DNA extraction, our method enhances the reliability of subsequent metagenomic analyses, providing a clearer and more detailed picture of the bacterial populations present on lettuce leaves.

## 3. Materials and Methods

### 3.1. Lettuce Cultivation Conditions

Soil was collected from an agricultural field regularly fertilized with manure, located in the Warmian–Masurian Voivodeship. After collection, the samples were transported to the laboratory and stored at 4 °C. Detailed soil characteristics are presented in [37]. Seeds of lettuce (*Lactuca sativa* L. var. Takoda) were sown in seedling pots measuring 24 × 24 mm. The plants were then grown for 4 weeks in climate chambers (POL-EKO, Wodzisław Śląski, Poland) at 18 °C/14 °C day/night, with a 16/8 h photoperiod and a light intensity of 8000 lx. Subsequently, 2 kg of soil was added to 17 cm diameter pots, and the lettuce seedlings were transferred (one seedling per pot). The plants were cultivated for another 4 weeks under the same conditions. After 4 weeks, the leaves were cut with sterile scissors 3 cm above the soil surface, mixed (to obtain a homogeneous sample), and analyzed.

### 3.2. Bacterial Cell Separation from Lettuce Leaves

To select the optimal method for separating cells from leaves, the following parameters of preliminary sample preparation were tested: the amount of starting material, the sonication step, the method of collecting bacterial cells (centrifugation or membrane filtration), and the preparation method of the membrane. Experimental variants were chosen based on a literature review using the keywords “phyllosphere”, “bacteria”, “DNA isolation”, and “16S RNA”. The scheme of the conducted experiments is shown in Figure 2. Lettuce leaves were prepared according to [38] with modifications.

#### Sample Preparation

An amount of 5 g (A) or 50 g of leaf (B) was placed in a glass bottle, and sterile 0.01 M PBS (phosphate-buffered saline) at pH 7.4 was added (45 mL per 5 g of leaf). Samples were sonicated (Sonic-3, 310 W, 40 KHz, POLSONIC Pałczyński, Warsaw, Poland) (C) for 7 min according to [38] or left without sonication (D). The bottles were then shaken for 1 h at 180 RPM on a laboratory shaker (DLAB Scientific, Senai, Malaysia) at 30 °C. To separate the leaf fragments, the bottle contents were filtered through gauze. Subsequently, 50 mL of the resulting liquid was centrifuged at 7500 RPM for 30 min (E) according to [18], or subjected to membrane filtration through sterile PVDF membranes with a 0.22 µm pore size and 47 mm diameter (Durapore, Merck Millipore, Burlington, MA, USA) (F), according to [38]. The pellets obtained during centrifugation and the membranes were stored at −80 °C until further analysis.

### 3.3. DNA Isolation

Each pellet was suspended in Tris-HCl buffer at pH 7, while PVDF membranes were cut into small fragments using sterile scissors (G) or left whole (H). DNA isolation was performed on the resulting pellet or PVDF membranes using the following commercially available kits: DNeasy Power Pro Soil Kit (Qiagen Inc.Germantown, MD, USA), which utilizes both mechanical and chemical lysis, and Genomic Mini AX Soil Spin (A&A Biotechnology, Gdansk, Poland), which uses enzymatic lysis (J), according to the manufacturers’ protocols. The experimental scheme is presented in Figure 2.

### 3.4. Experimental Variants, Quantity, and Quality of Isolated DNA

The planned experiments were conducted with 24 variants (Table 2). The quantity of isolated DNA was measured using a NanoDrop 1000 spectrophotometer (NanoDrop Technologies, Wilmington, NC, USA). The purity of the isolated DNA was assessed using the OD 260/280 and OD 260/230 parameters (NanoDrop Technologies, Wilmington, NC, USA).

### 3.5. Next-Generation Sequencing

To confirm that the isolated genetic material originated from bacteria, next-generation sequencing (NGS) was performed. The metagenomic analysis of the phyllosphere samples was conducted by an external company (Genomed S.A., Warsaw, Poland). The bacterial population analysis was based on the hypervariable V3-V4 region of the 16S rRNA gene. Specific primer sequences 341F and 785R were used for the amplification of the selected region and library preparation. The PCR reaction was carried out using the Q5 Hot Start High-Fidelity 2X Master Mix (New England Biolabs Inc., Ipswich, MA, USA), following the manufacturer’s recommendations. Sequencing was performed on a MiSeq instrument using paired-end (PE) 2 × 300 nt technology with the Illumina v3 kit. Bioinformatic analysis, ensuring taxonomic classification, was conducted using the QIIME 2 software package (version 2017.6.0) based on the Silva 138 reference sequence database. The DADA2 package was also employed to distinguish biological origin sequences from those newly formed during the sequencing process. This package was additionally used to extract sequences of unique biological origin, known as amplicon sequence variants (ASVs).

### 3.6. Statistical Analyses

All experiments were conducted in 10 replicates. One-way ANOVA with Tukey’s post hoc test was used to assess the differences in DNA concentration between the extraction methods. Statistical analyses were performed using GraphPad Prism 8.0.1.

## 4. Conclusions

In this work, we achieved the optimization of our DNA extraction protocol, including specific steps such as mechanical–chemical lysis, sonication, and membrane filtration. These steps were designed not only to maximize bacterial DNA yield, but also to selectively target bacterial cells and reduce the carryover of plant DNA. The success of these techniques in reducing chloroplast DNA contamination while retaining high bacterial DNA integrity and quantity is crucial for downstream applications, such as metagenomic sequencing and microbial diversity studies. Overall, the effective limitation of chloroplast DNA co-extraction and the high percentage of bacterial DNA obtained reflect the methodological rigor and the potential applicability of our approach to other plant microbiome studies. This method can serve as a reliable protocol for researchers aiming to study the complex interactions within the phyllosphere microbiome, ensuring that the focus remains on microbial communities rather than being confounded by plant DNA.

## Figures and Tables

**Table 1:** Effects of lysis method (enzymatic, mechanical–chemical) and extraction conditions on yield [ng/µL], total yield [ng], and purity [A_260/280_, A_260/230_] of bacterial DNA isolated from lettuce phyllosphere. Small letters represent groups of significant difference.

Number of Experiment	DNA [ng/µL]	DNA [ng]	A260/280	A260/230
Enzymatic lysis				
1	3.94 ± 0.70 a	194 ± 39.4 a	1.40 c	0.77 b
2	6.41 ± 0.95 b	327 ± 45.0 b	1.50c	1.10d
3	7.45 ± 0.95 b	378 ± 47.0 b	1.48 c	0.97 c
4	3.14 ± 0.69 a	160 ± 35.8 a	1.20 a	0.75 b
5	4.86 ± 1.02 b	241± 53.9 b	1.34 b	1.00 c
6	4.19 ± 1.03 a	206 ± 53.7 a	1.50 b	1.11 d
7	5.56 ± 1.19 b	271 ± 59.0 b	1.30 b	0.70 b
8	10.30 ± 1.38 c	521 ± 70.5 c	1.60 d	1.40 e
9	11.50 ± 1.00 c	575 ± 54.7 c	1.53 c	1.13 d
10	5.57 ± 0.70 b	281 ± 36.4 b	1.30 b	0.57 a
11	6.90 ± 0.65 b	348 ± 32.9 b	1.40 b	0.73 b
12	6.99 ± 1.16 b	346 ± 60.5 b	1.54 c	0.77 b
Mechanical–chemical lysis				
13	6.07 ± 0.81 b	306 ± 42.0 b	1.60 d	1.12 d
14	9.50 ± 1.35 c	470 ± 69.8 c	1.75 f	1.79 g
15	9.98 ± 1.20 c	490 ± 57.4 c	1.90 g	1.50 f
16	5.48 ± 0.69 b	276± 35.8 b	1.68 f	1.45 f
17	6.81 ± 0.84 b	345 ± 42.5 b	1.85 g	1.78 g
18	6.84 ± 0.75 b	342 ±39.6 b	1.90 g	1.62 g
19	5.67 ± 0.84 b	280 ± 43.2 b	1.78 f	1.56 f
20	24.29 ± 4.32 d	1205 ± 226.6 d	1.83 g	1.56 f
21	38.08 ± 4.28 f	1929 ± 210.4 f	1.85 g	1.81 g
22	5.84 ± 0.93 b	301 ± 37.7 b	1.76 f	1.74 g
23	8.41 ± 1.39 c	420 ± 73.9 c	1.84 g	0.91 c
24	9.20 ± 0.93 c	461 ± 49.6 c	1.90 h	1.87 h

**Table 2:** Overview of experiments for bacterial DNA isolation from lettuce phyllosphere.

Number of Experiment	Experimental Steps
1	5 g of lettuce leaf, sonication, centrifugation, enzymatic lysis
2	5 g of lettuce leaf, sonication, membrane filtration, entire membrane, enzymatic lysis
3	5 g of lettuce leaf, sonication, membrane filtration, cut membrane, enzymatic lysis
4	5 g of lettuce leaf, without sonication, centrifugation, enzymatic lysis
5	5 g of lettuce leaf, without sonication, membrane filtration, entire membrane, enzymatic lysis
6	5 g of lettuce leaf, without sonication, membrane filtration, cut membrane, enzymatic lysis
7	50 g of lettuce leaf, sonication, centrifugation, enzymatic lysis
8	50 g of lettuce leaf, sonication, membrane filtration, entire membrane, enzymatic lysis
9	50 g of lettuce leaf, sonication, membrane filtration, cut membrane, enzymatic lysis
10	50 g of lettuce leaf, without sonication, centrifugation, enzymatic lysis
11	50 g of lettuce leaf, without sonication, membrane filtration, entire membrane, enzymatic lysis
12	50 g of lettuce leaf, without sonication, membrane filtration, cut membrane, enzymatic lysis
13	5 g of lettuce leaf, sonication, centrifugation, mixed lysis
14	5 g of lettuce leaf, sonication, membrane filtration, entire membrane, mixed lysis
15	5 g of lettuce leaf, sonication, membrane filtration, cut membrane, mixed lysis
16	5 g of lettuce leaf, without sonication, centrifugation, mixed lysis
17	5 g of lettuce leaf, without sonication, membrane filtration, entire membrane, mixed lysis
18	5 g of lettuce leaf, without sonication, membrane filtration, cut membrane, mixed lysis
19	50 g of lettuce leaf, sonication, centrifugation, mixed lysis
20	50 g of lettuce leaf, sonication, membrane filtration, entire membrane, mixed lysis
21	50 g of lettuce leaf, sonication, membrane filtration, cut membrane, mixed lysis
22	50 g of lettuce leaf, without sonication, centrifugation, mixed lysis
23	50 g of lettuce leaf, without sonication, membrane filtration, entire membrane, mixed lysis
24	50 g of lettuce leaf, without sonication, membrane filtration, cut membrane, mixed lysis

## Data Availability

The original contributions presented in the study are included in the article.
